# Effects of Hermetic Storage on Adult *Sitophilus oryzae* L. (Coleoptera: Curculionidae) Acoustic Activity Patterns and Mortality

**DOI:** 10.1093/jee/tox260

**Published:** 2017-10-16

**Authors:** A W Njoroge, R W Mankin, B W Smith, D Baributsa

**Affiliations:** 1 Department of Entomology, Purdue University; 2 United States Department of Agriculture, Agricultural Research Service Center for Medical, Agricultural and Veterinary Entomology

**Keywords:** detection, oxygen, insect activity, metabolism, hermetic storage

## Abstract

Hermetic storage is of interest to farmers and warehouse managers as a method to control insect pests in small storage facilities. To develop improved understanding of effects of hermetic storage on insect pest activity and mortality over time, oxygen levels, acoustic signals, and observations of visual movement were recorded from replicates of 25, 50, and 100 adult *Sitophilus oryzae* (L.) (Coleoptera: Curculionidae) hermetically sealed in 500- and 1,000-ml glass jars. Recordings were done for 28 d; twice daily for the first 6 d and twice weekly thereafter. Insect sounds were analyzed as short bursts (trains) of impulses with spectra that matched average spectra (profiles) of previously verified insect sound impulses. Oxygen consumption was highest in treatments of 100 insects/500-ml jar and lowest in 25/1000-ml jars. The rates of bursts per insect, number of impulses per burst, and rates of burst impulses per insect decreased as the residual oxygen levels decreased in each treatment. Activity rates <0.02 bursts s^−1^, the acoustic detection threshold, typically occurred as oxygen fell below 5%. Mortality was observed at 2% levels. The time to obtain these levels of insect activity and oxygen depletion ranged from 3–14 d depending on initial infestation levels. Acoustic detection made it possible to estimate the duration required for reduction of insect activity to levels resulting in negligible damage to the stored product under hermetic conditions. Such information is of value to farmers and warehouse managers attempting to reduce pest damage in stored crops.

Hermetic storage has been of longstanding interest as a physical method for control of postharvest insect pests (Bailey 1955, Moreno-Martinez et al. 2000). One method of control is to remove or replace atmospheric oxygen (O_2_) in the storage enclosure (Adler et al. 2000, Hoback and Stanley 2001, Navarro 2006). A second method, the use of sealed, gas-impervious hermetic enclosures, is of increasing interest in locales where high levels of infestation are prevalent in small-scale storage facilities (Tefera et al. 2011, Murdock et al. 2012, De Groote et al. 2013, Martin et al. 2015, Williams et al. 2017). In the latter method, the hermetic enclosures seal commodities so tightly that respiration of aerobic organisms in the commodities depletes O_2_ enough to cause mortality.

Several studies have been conducted to determine the timing of mortality under controlled atmospheres with reduced O_2_ or added carbon dioxide. Bailey (1955) found, for example, that a mixture of 40% carbon dioxide and 2% oxygen for 17 d was required to achieve 100% mortality of adult and immature *Calandra granaria* L (Coleoptera: Dryophthoridae) (Bailey 1955). In sealed hermetic environments, the drop in O_2_ is driven by natural processes and depends on insect species present, pest population, and initial amount of O_2_ available. Moreno-Martinez et al. (2000) found that all *Sitophilus zeamais* L. (Coleoptera: Curculionidae) were dead after 12-d exposure to hermetic conditions where oxygen was reduced to 0% after 6 to 9 d. However, the mechanisms and temporal pattern of insect physiological and behavioral decline and subsequent death during hermetic storage are not well characterized.

Several studies have sought specific mechanisms that cause insect mortality in hermetic storage. Bailey (1955) measured respiratory quotient (ratio of CO_2_ produced to O_2_ consumed) of insects in airtight conditions. Death was due to the depletion of O_2_ (caused by the respiration of the insects and the grain) rather than the accumulation of CO_2_ (Bailey 1955). Other studies have found that death is due to desiccation rather than suffocation (Murdock et al. 2012).

Previous studies have shown the effectiveness of acoustic technology in monitoring insect feeding and movement activity and estimating population levels in a grain mass in experimental bins and commercial silos (Hagstrum et al. 1988, 1991, 1994), as well as in the laboratory (Shade et al. 1990, Mankin et al. 1997, Murdock et al. 2012). Other investigations of *Sitophilus oryzae* L. (Coleoptera: Curculionidae) with acoustic methods have been conducted in grain bins to assess detectability of infestations under different temperature and pest density conditions (Fleurat-Lessard et al. 2006). These studies showed that the rates of insect sounds increased with pest density. In general, sounds produced by stored product insects consist of bursts (trains) of brief impulses with average spectra (profiles) that are similar for a given species feeding at a given time on a given substrate (Pittendrigh et al. 1997, Mankin et al. 2011, Kiobia et al. 2015). Customized software can be used to identify impulse trains as insect sound bursts or nontarget background noise by matching their spectra against spectral profiles of known insect sounds (Mankin et al. 2011). The rate of insect sound bursts detected in a given treatment can be used to estimate the likelihood of infestation (Mankin et al. 2011, Dosunmu et al. 2014). A minimum rate of 0.02 bursts s^−1^ was used as a detection threshold below which an insect is not detectable above background or the sample is considered uninfested (Mankin et al. 2008, Njoroge et al. 2016). Acoustic signal analysis enables monitoring of insect activity during hermetic storage and estimation of durations required for reduction of activity to levels resulting in negligible damage to the stored product.

The acoustic monitoring study was conducted with *S. oryzae*. This insect and its close relatives, *S. zeamais* L. and *Sitophilusgranarius* L. (Coleoptera: Curculionidae), are regarded as insect pests of economic importance in stored rice, wheat, barley, sorghum, and maize (Plarre 2010). In tropical climates, *S. zeamais* infestations often begin in mature crops in fields before harvest (e.g., Adedire 2001).

For this study, we employed O_2_ sensors, acoustic sensors, and visual observations to measure activity as well as mortality of *S. oryzae* over a 28-d period as O_2_ was depleted in hermetic environments containing different numbers of adults. The objective of this study was to quantify the declining activity of different populations of *S. oryzae* as oxygen depletes in different hermetic environments and establish when insects cease feeding at economically damaging levels.

## Materials and Methods

### Insects

Unsexed *S. oryzae* adults were obtained from laboratory colonies maintained in a Conviron Environmental Chamber (C710, Winnipeg, MB, Canada) at the Department of Entomology, Purdue University. The *S. oryzae* were reared on wheat at 28 ± 1ºC; 65 ± 5% RH on a 12:12 (L:D) photoperiod. For each experiment, adult *S. oryzae* were isolated from the colony using a No. 20 Sieve. The collected insects were then counted via vacuum aspiration into groups of 25, 50, or 100 for introduction into specific hermetic treatments.

### Hermetic Storage Jars

The experiments were carried out in an isolated quiet room at 25 ± 1ºC. Each treatment had three replications in round reusable Pyrex 1,000 and 500-ml glass jars (Corning Inc., Germany). To monitor O_2_, each of the jars was fitted with two OxyDots (Oxysense Inc, Dallas, TX) 1 d prior to the start of the experiments to allow the glue to set. The jars were sealed with size No. 7 rubber stoppers after 500 g of wheat were placed in each 500-ml jar and 1,000 g in each 1,000-ml jar. A hole was drilled through each stopper using a cordless drill (Black & Decker (US), Towson, MD) fitted with a 19/64-inch drill bit (Menards Inc, Eau Claire, WI). A stainless steel probe then was fitted through the drilled hole to serve as a waveguide for transmission of vibrational signals to the acoustic sensor-amplifier system. Data loggers (EL-USB-2, Lascar Electronics Inc., PA) were set to record temperature and RH every 30 min.

To estimate the initial volumes of oxygen available for the insects to consume in the different-sized jars, we added water to three wheat grain-filled jars using a beaker. The quantity of water added in the intergranular space in the grain-filled jar (removed from the beaker) was used as an estimate of the air volume. By this procedure, the volume of air available in the headspace and intergranular space of the wheat-filled 1000-ml jars was estimated to be 480 ml, while that of the 500-ml jars was 260 ml. Because O_2_ makes up about 21% of the atmospheric air, the amount of O_2_ available at the beginning of the experiment was estimated to be 53 and 95 ml in the 500- and 1,000-ml jars, respectively.

### Grain Preparation and Infestation

Clean wheat for the experiments was sourced from Purdue Farms (West Lafayette, IN). To ensure there was no existing infestation, it was first stored 14 d at −18ºC and then set out at room temperature 1 d before the start of the experiment. The clean grain then was poured into nine 500- and 1,000-ml jars each, and three replicates each of the precounted groups of 25, 50, and 100 adult *S. oryzae* were introduced separately into the jars.

### Acoustic, Visual, and Oxygen Monitoring and Recording

The setup for monitoring and recording insect signals was similar to that described in Herrick and Mankin (2012). A sensor-preamplifier module (model SP-1L Acoustic Emission Consulting [AEC], Sacramento, CA) was attached at the end of the waveguide (probe) passing through the sealing cork into the infested grain in the jars. The sensor was connected to an AED 2010 amplifier (AEC, Sacramento, CA). The AED-2010 was connected to a digital audio recorder, either a Tascam [model HD-P2, Montebello, CA] or Marantz professional [model PMD-561, New York City, NY], both of which stored the insect signals as .wav files on memory cards at the same 44.1 kHz sampling rate. The laboratory was located in a secluded area with minimal background noise interference. Recordings of 1-h each were taken twice a day (morning and evening) for the first 6 d and twice a week for the next 22 d as the activity decreased. The OxyDot levels were checked visually immediately before acoustic recordings, and insects observable through the glass walls of the jars were checked for presence of normal, weak, or no visible movement.

### Acoustic Signal Analysis

Signals were prescreened using Raven Lite software (Bioacoustics Research Program 2016), and a custom-written insect signal analysis software program DAVIS (Digitize, Analyze, View, Insect Sounds) (Mankin 1994, Mankin et al. 2000, Herrick et al. 2013) performed analyses of a 77- to 300-s sample selected at random from each recording to distinguish insect sound impulses from occasionally occurring background noise. Movement and feeding sounds of insects in stored products generally occur as trains of brief, 1- to 10-ms impulses separated by <200 ms intervals (Njoroge et al. 2016). For each analyzed section, the DAVIS program classified these individual sound impulses as insect signals or background noise by least squares matching of their spectra against spectral profiles of known insect sounds (Mankin et al. 2011). In this experiment, we matched the impulse spectra against two different representative profiles, both from recordings of separate infestations observed in a preliminary study. One profile was an average of 139 impulses detected over a 62-s interval, and the second profile was an average of 33 impulses detected over a 20-s interval. Impulse trains that contained at least three impulses which matched one of the two profiles were categorized as insect sound bursts (Jalinas et al. 2015; Njoroge et al. 2016, 2017). If the spectrum of a given impulse did not match one of the two insect sound impulse profiles, the impulse was classified as background noise and discarded from further analysis. The times and types of individual impulses and insect sound bursts in each file were saved in a spreadsheet for analysis of three different acoustic measures of insect activity: mean burst rates (*R*_*b*_), mean counts of impulses per burst (*N*_*b*_), and mean rates of burst impulses (*R*_*bimp*_), i.e., rates of impulses detected only within bursts (Jalinas et al. 2015).

### Assessment of Grain Prehermetic and Post-hermetic Storage

At the beginning and end of the experiment, the moisture content, weight loss, and germination capacity of wheat sampled from the grain used in each experimental test was measured. Moisture content was determined using a handheld grain moisture tester; Dickey-John mini GAC plus moisture tester (DICKEY-john Corporation, IL).

For weight loss assessment, 125-g subsamples were taken from each treatment and separated into damaged and undamaged grain portions taking the weight and count of each portion (Boxall 1986). Percentage weight loss was then determined using equation 1:

$$100\times \frac{\text{ND(WU)}-\text{NU(WD)}}{\text{WU}(\text{NU}+\text{ND})}$$ (1)

where WU is weight of undamaged grains, WD weight of insect damaged grains, NU number of undamaged grains, and ND is the number of insect damaged grains.

Germination capacity was determined as described by Baoua et al. (2014). Four subsamples of 25 wheat grains were randomly selected from each treatment and placed in four petri dishes lined with filter paper. Water was added to moisten the filter paper and the petri dishes left for 7 d and the number of germinated seeds counted.

### Statistical Analyses

All data were analyzed using Stata SE Version 12 (Stata Corp, TX) or regression analysis (Proc GLM, SAS Institute 2012) Version 9.4. The residual oxygen percentage and insect activity measurements were subjected to analysis of variance (ANOVA). Analysis of covariance (ANCOVA) was applied to test effects of treatment, storage duration, and the interaction of treatment and storage duration. For insect activity, the coefficient of the interaction term was significant (*P* ≤ 0.05) and therefore one-way ANOVA was performed to test for daily differences during the first 5 d of storage. Means were separated using Bonferroni adjustment at 95% confidence level. The regression of mean activity rate on mean residual O_2_ percentage was analyzed. Pre-storage and post-storage quality grain parameters of moisture content, weight loss, and germination capacity were subjected to one-way ANOVA.

## Results

### Oxygen Depletion Trends for Different Treatments

The daily OxyDot readings were used to estimate the residual levels of O_2_ over time in jars with different treatments. ANCOVA showed statistically significant differences in mean residual O_2_ percentages among treatments (*F*_5, 647_ = 57.03; *P* < 0.001) at different storage times (*F*_12, 647_ = 221.51; *P* < 0.001) and their interaction (*F*_41, 647_ = 13.79; *P* < 0.001). The significance of the interaction term indicated that each treatment showed significant differences in the rate of decline of O_2_ throughout the storage period; consequently, ANOVA was carried out to determine the statistical significance of differences on days 1, 5, 15, and 25 (Table 1). On the first day, the effect of treatment on residual O_2_ percentage was not significant, but means in both 25-insect treatments were significantly different from those in other treatments by day 5, and multiple significant differences among treatments were observed on subsequent days.

**Table 1.** Analysis of variance of residual oxygen level in 25, 50, 100 adults of *S. oryzae* on days 1, 5, 15, and 25 of hermetic storage treatment in 500-ml and 1,000-ml jars (*n* = 162)

Treatment	Residual percentage of O_2_ (mean ± SEM)*			
	Day 1	Day 5	Day 15	Day 25
25 insects/1,000 ml	19.84 ± 0.08a	12.16 ± 0.75a	3.55 ± 1.00a	2.72 ± 0.21a
25 insects/500 ml	20.41 ± 0.17a	11.69 ± 0.44a	2.20 ± 0.16b	2.36 ± 0.02b
50 insects/1,000 ml	19.73 ± 0.13a	9.89 ± 0.52b	2.07 ± 0.07b	1.84 ± 0.09c
50 insects/500 ml	20.36 ± 0.35a	7.54 ± 0.81c	0.41 ± 0.27c	0.40 ± 0.09d
100 insects/1,000 ml	19.70 ± 0.72a	3.57 ± 1.60d	0.85 ± 0.20c	0.72 ± 0.25d
100 insects/500 ml	20.19 ± 0.69a	0.75 ± 0.22e	0.23 ± 0.04c	0.21 ± 0.02d

*All data are means ± standard error of mean (SEM). Entries in the same column followed by same letters are not significantly different (*P* > 0.05). Means were separated using Bonferroni adjustment.

**Fig. 1.**
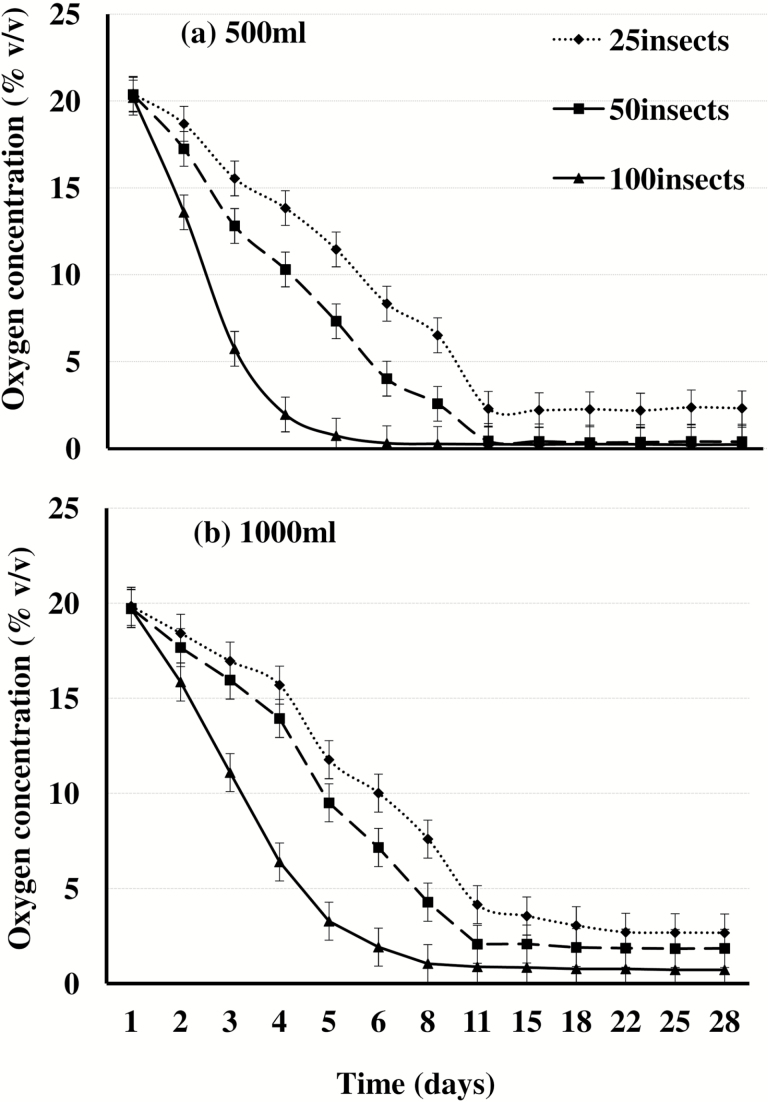
Oxygen consumption patterns for 25, 50, and 100 *Sitophilus oryzae* adults enclosed in hermetically sealed (a) 500-ml jars and (b) 1,000-ml jars.

The total O_2_ volume consumed after 28 d was computed and used to calculate the quantity of O_2_ consumed per insect in different treatments (Table 2). As expected, residual O_2_ percentages decreased most rapidly in the 500-ml jars with 100 insects, while they decreased most slowly in the 1,000-ml jars with 25 insects (Fig. 1). In all treatments, most of the reductions occurred by the 11th day.

**Table 2.** Estimated (Est) initial and final O_2_ volumes and volume consumption per insect after 28 d in different hermetic treatments

Treatment	Est initial O_2_ volume (ml)*	Est final O_2_ volume (ml)*	Est consumed O_2_/insect (ml)*
25 insects/1,000 ml	95.2 ± 0.06	12.6 ± 0.12	3.3 ± 0.01
25 insects/500 ml	53.1 ± 2.94	6.0 ± 1.15	1.9 ± 0.07
50 insects/1,000 ml	94.7 ± 2.71	8.8 ± 1.50	1.7 ± 0.02
50 insects/500 ml	52.9 ± 2.25	1.0 ± 0.23	1.0 ± 0.04
100 insects/1,000 ml	94.5 ± 3.72	3.5 ± 0.75	0.9 ± 0.03
100 insects/500 ml	52.5 ± 2.63	0.6 ± 0.29	0.5 ± 0.02

*All data are means ± standard error of mean (SEM).

### Sound Burst Rate Trends for Different Treatments

**Fig. 2.**
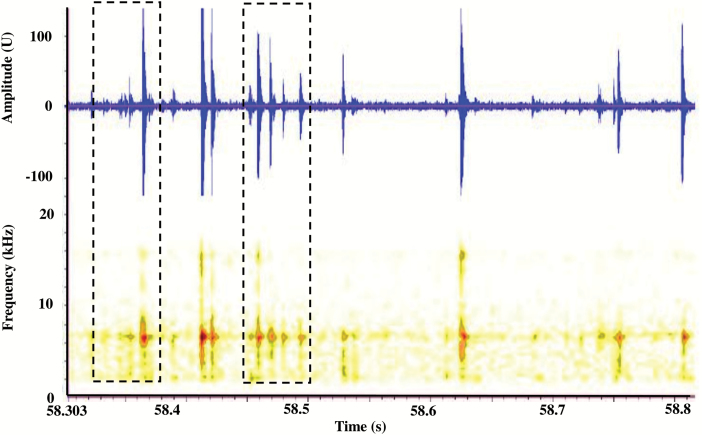
Oscillogram and spectrogram, of a 0.5-s period of impulse patterns recorded from wheat infested with 100 adults of *S. oryzae* contained in a 1000-ml Pyrex glass jar immediately after sealing with a rubber stopper. Activity of higher energy is denoted by darker shading on the spectrogram (256 points per spectrum, 50% overlap).

**Fig. 3.**
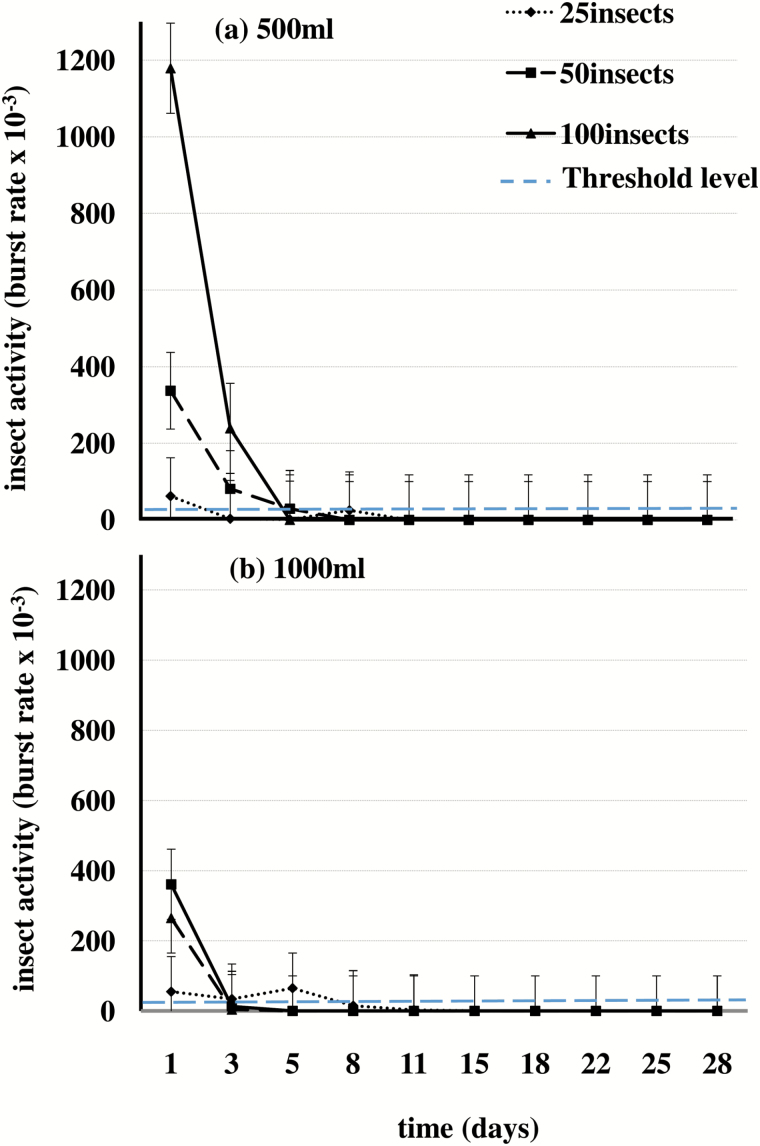
Rates of sound bursts from 25, 50, and 100 *S. oryzae* adults enclosed in hermetically sealed (a) 500-ml jars and (b) 1,000-ml jars. The dashed line indicates the 0.02 bursts s^−1^ threshold level for low likelihood of insect presence.

For the first 5 d after onset of storage treatments, signals identifiable as trains (bursts) of insect sound impulses with a broad range of amplitudes, spectral features, and temporal patterns were detected frequently in each treatment. Fig. 2 is an example showing a typical range of signals in a 0.5-s section of recording from a jar infested with 100 insects. Several groups (trains) of impulses separated by < 200 ms occur are seen in the example, including those in the intervals of 58.32–58.38 s and 58.45–58.50 s. The spectra of the impulses in these trains matched well with profiles of previously verified insect sounds (as discussed in Methods above); consequently, the trains were considered to be insect sound bursts. In all treatments, the rates of signals identified as insect sound bursts were proportional to the numbers of insects per jar and the rates were highest at the onset of treatment (Fig. 3). The burst rates thereafter decreased, falling below the threshold levels of 0.02 bursts s^−1^ between 3–8 d, depending on the treatment (Fig. 3).

The burst rates in all treatments decreased steadily until 5% O_2_ was attained in the jars. After declining to 5%, few bursts were recorded and the activity by insects that were observable through the glass declined from normal to occasional weak movements. Within 3 d after reaching 5%, the level in all treatments declined further to 2% or lower. At 2%, no insect activity was observed and the insects were presumed dead. The time taken to attain 2% O_2_ varied from 5 to 13 d among treatments (Table 3).

**Table 3.** Time after onset until reduction to 5 and 2% residual oxygen, and rate of oxygen depletion to 2% level for different treatments

Treatment	Mean time (d) ± SEM to reach		Rate of oxygen depletion (population/d)^*a*^
	5% oxygen level	2% oxygen level	
25 insects/1,000 ml	10.5 ± 0.17	13 ± 2.31	1.92 ± 0.09
25 insects/500 ml	8.5 ± 0.35	11 ± 0.58	2.27 ± 0.12
50 insects/1,000 ml	7.8 ± 0.87	12 ± 1.73	4.17 ± 0.20
50 insects/500 ml	5.9 ± 0.69	8 ± 2.31	6.25 ± 0.46
100 insects/1,000 ml	4.5 ± 0.52	6 ± 1.73	16.67 ± 1.66
100 insects/500 ml	3.1 ± 0.75	5 ± 1.15	20 ± 2.42

^*a*^Rate of oxygen depletion was calculated as the population in the treatment jar divided by the number of days until depletion to 2% O_2_.

ANCOVA was conducted to test effects of treatment and storage durations and their interaction on the mean rates of bursts, mean No. impulses per burst, and mean rates of burst impulses obtained from the DAVIS analysis (Table 4). The *F* values were statistically significant for the mean rates of bursts, impulses per burst, and rates of burst impulses. To further evaluate effects over storage durations, we then performed one-way ANOVA to compare mean burst rates from different treatments over the first 5 d (Table 5). There was a greater significant difference among treatments during the first 3 d when the insects were most active, and by the fifth day there was no significant difference among the treatments.

**Table 4.** Analysis of covariance of effects of hermetic storage treatment, storage duration, and their interaction on the mean rates of bursts, numbers of impulses per burst, and rates of burst impulses (*n* = 648 observations)

Parameter	Df	*F*	*P*
Rates of bursts			
Treatment	5	11.77	<0.001
Storage duration	12	17.64	<0.001
Treatment × storage duration	41	2.76	<0.001
Impulses per burst			
Treatment	5	3.79	0.0025
Storage duration	12	12.42	<0.001
Treatment × storage duration	41	1.78	0.004
Rates of burst impulses			
Treatment	5	8.46	<0.001
Storage duration	12	16.77	<0.001
Treatment × storage duration	41	1.91	0.001

**Table 5.** Analysis of variance of insect sound burst rates produced by 25, 50, and 100 *Sitophilus oryzae* adults during the first 5 d of hermetic storage treatment in 500-ml and 1,000-ml jars (*n* = 324)

Treatment	Daily *Sitophilus oryzae* mean activity (bursts s^−1^)*				
	Day 1	Day 2	Day 3	Day 4	Day 5
25 insects/500 ml	0.04 ± 0.01a	0.01 ± 0.01a	0 ± 0a	0.09 ± 0.05a	0 ± 0a
25 insects/1,000 ml	0.37 ± 0.16bc	0.05 ± 0.01a	0.02 ± 0.01a	0 ± 0a	0 ± 0.03a
50 insects/500 ml	0.23 ± 0.05a	0.18 ± 0.07b	0.05 ± 0.03ab	0.1 ± 0.04a	0.02 ± 0.01a
50 insects/1,000 ml	0.24 ± 0.04a	0.05 ± 0.01a	0.01 ± 0.01a	0 ± 0a	0 ± 0a
100 insects/500 ml	0.79 ± 0.17c	0.08 ± 0.02a	0.16 ± 0.06b	0 ± 0a	0 ± 0a
100 insects/1,000 ml	0.18 ± 0.01ab	0 ± 0	0.01 ± 0.01a	0.03 ± 0.01a	0 ± 0a

*All data are means ± SEM. Entries in the same column followed by same letters are not significantly different (*P* ≥ 0.05). Means were separated using Bonferroni adjustment.

### Regressions of Acoustic Activity Level on Oxygen Level

**Fig. 4.**
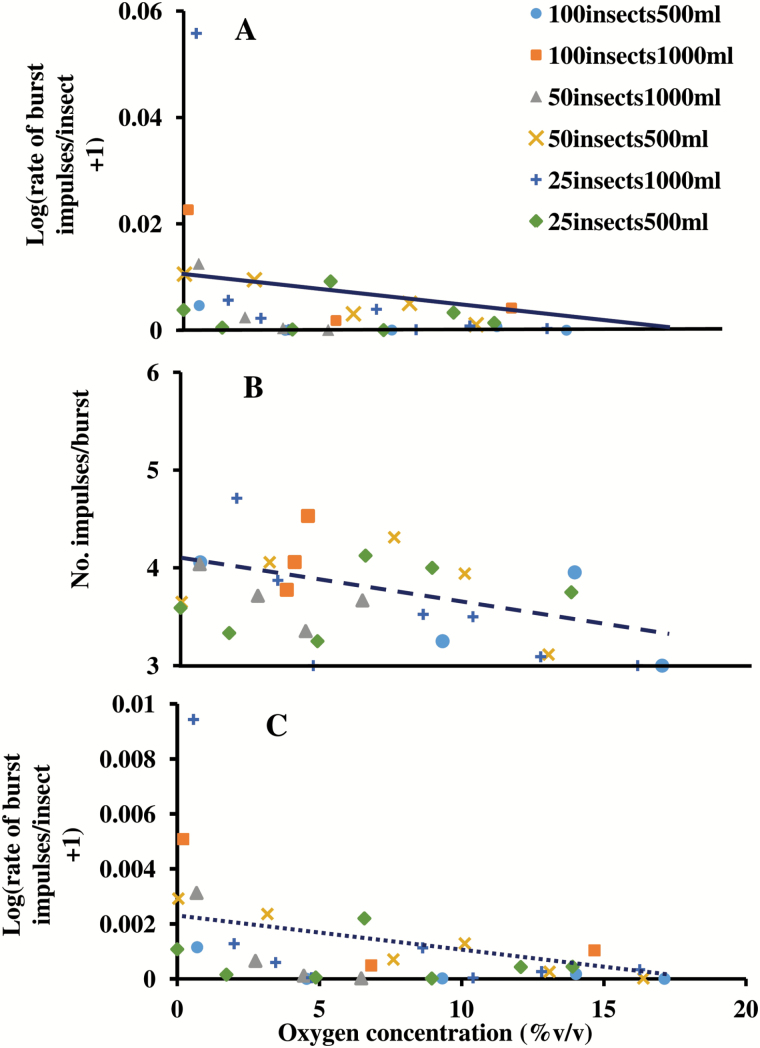
Acoustic activity at different O_2_ percentages in hermetic treatments measured as: (A) rate of burst impulses, *R*_*bimp*_, normalized per No. insects per treatment, *N*_*t*_, (*R*_*bimp*_*/N*_*t*_), (B) No. impulses per burst, and (C) rate of bursts, *R*_*b*_, normalized per No. insects per treatment (*R*_*b*_*/N*_*t*_). Horizontal scale is % O_2_ and treatment mean values at each O_2_ value are designated as: 25 insects/500 ml, diamond; 25 insects/1,000 ml, plus; 50 insects/500 ml, X; 50 insects/1000 ml, triangle; 100 insects/500 ml, square; and 100 insects/1,000 ml, circle. Vertical scale units are (A) 0.01, (B) 1, and (C) 0.01. Vertical scale values are: (A) Log_10_ (*R*_*bimp*_/*N*_*t*_+1); (B) No. impulses per burst; and (C) Log_10_(*R*_*b*_*/N*_*t*_+1). Regressions are shown as (A) solid line, (B) dashed line, and (C) dotted line.

It was of interest from a physiological perspective to consider how different measures of acoustic activity were affected by O_2_ levels across hermetic treatments. The rates of bursts, counts of impulses per burst, and rates of burst impulses were expected to decrease as the O_2_ levels decreased because the energy available to move rapidly or forcefully was decreasing. To conduct such analyses, the measures of acoustic activity calculated from recordings of each treatment first were plotted in several formats to consider the type of display in which treatment effects could be interpreted most easily. The values for *N*_*b*_ already were scaled in terms of the number of bursts per file, and the range was constrained narrowly between 3 and 6 impulses per burst; consequently, it was feasible to display the values of *N*_*b*_ of all treatments directly against the residual O_2_ percentage, as in Fig. 4B. However, to plot the activity rates *R*_*b*_ and *R*_*bimp*_ from different treatments on a normalized scale, e.g., rate of bursts per insect, the rates were divided first by the number of insects in each treatment, *N*_*t*_. In addition, due to their wide ranges of magnitudes, the rescaled values, *R*_*b*_*/N*_*t*_ and *R*_*bimp*_*/N*_*t*_, were plotted using a Log_10_ (magnitude +1) transformation for ease of interpretation in Fig. 4A and Fig. 4C, respectively. It was found that the horizontal axis could be interpreted easily either in terms of residual O_2_ percentage, as shown in Fig. 4, or as O_2_ depletion, where depletion = (initial – residual) oxygen percentage from Table 2.

Inspection of the combined data points from all treatments in Fig. 4 suggested the testing of three statistical models to describe the trend of activity magnitudes against depletion:

$${\text{Log}}_{\text{1}0}(R_{b}\text{/}N_{t}+\text{1})=\text{depletion}$$ (2)

$$N_{b}=\text{depletion}$$ (3)

$${\text{Log}}_{\text{1}0}(R_{bimp}\text{/}N_{t}+\text{1})=\text{depletion}$$ (4)

The models are statistically significant, with *F*_1,32_ = 9.45 (*P* = 0.0043) with *R*^2^ = 0.228 for equation 2, *F*_1,28_ = 6.42 (*P* = 0.0172) with *R*^2^ = 0.186 for equation 3, and *F*_1,30_ = 6.18 (*P* = 0.0188) with *R*^2^ = 0.171 for equation 4. The intercepts and slopes of the regression equations are significantly different from zero [*P* (>*t*) < 0.05] (Table 6). The regression lines are shown in Figs. 4A (solid line), Figs. 4B (dashed line), and Figs. 4C (dotted line). As expected, the values of *R*_*b*_*/N*_*t*,_, *N*_*b*_, and *R*_*bimp*_*/N*_*t*_ all decreased with decreasing residual O_2_ percentage.

**Table 6.** Intercepts and slopes (± SEM) (all values ×10^–3^) for regression equations fitting the models in equations 2–4

Measurement	Intercept ± SEM	*t*	*P > t*	Slope ± SEM	*t*	*P > t*
*R*_*b*_ (equation 2)	2.26 ± 0.477	4.74	<0.001	−0.156 ± 0.0507	−3.07	<0.001
*N*_*b*_ (equation 3)	4,132 ± 176	23.6	<0.001	−50.87 ± 20.08	−2.53	0.0172
*R*_*bimp*_ (equation 4)	10.8 **±** 2.8	3.81	0.0006	−0.818 **±** 0.329	−2.49	0.0188

**Fig. 5.**
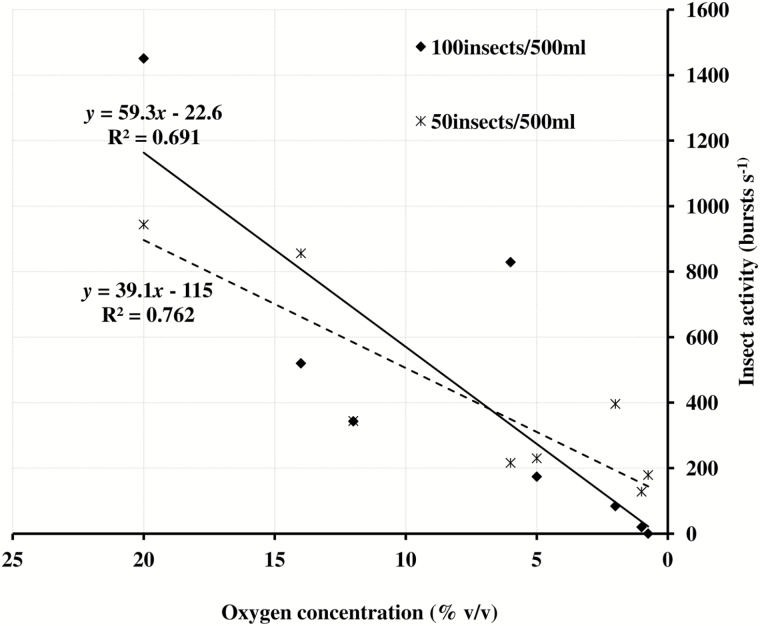
Regression analysis of oxygen consumption and insect activity for 50 insects/500 ml and 100 insects/500 ml treatments.

The *R*^2^ values of the regression lines in Fig. 4 possibly were negatively affected by the significant interaction between treatment and storage duration (Table 5). To consider such an effect, regression analysis was performed separately on two individual treatments, 50 insects/500 ml and 100 insects/1,000 ml. A direct linear relationship was effective in explaining the decline in sound activity as O_2_ declined (Fig. 5). The fitted equations were: *y* = 59.3*x* – 22.6 and *y* = 39.1*x* + 115 and the estimated error variances were *s*^2^*=* 300 and *s*^2^ = 166, with corresponding standard deviations of *s* = 17.32 and *s* = 12.88, for the 100-insect and 50-insect treatment, respectively. The coefficients of determination (*R*^2^) for the regression curves of burst rate against O_2_ level for 50 insects/500 ml and 100 insects/1,000 ml treatments were 0.6913 and 0.7618, respectively, as shown in Fig. 5, much higher than in Fig. 4, where all treatments were combined.

### Assessment of the Grain Prehermetic and Post-hermetic Storage

After 28 d, the jars were opened and no surviving adults, eggs, or larvae were present in the wheat. Samples were analyzed for moisture content, percentage weight loss and germination capacity as described in the Materials and Methods. Before storage, the moisture content was determined to be 8% since the wheat was prepared for long-term storage. At the end of the 28-d storage period, there was no significant difference (*F*_5, 17_ = 1.26; *P* = 0.3414) in grain moisture between the six treatments (Table 7). After 28 d of hermetic storage under different levels of insect infestation; the weight loss values were approximately 1%, and there was no significant difference (*F*_5, 17_ = 0.38; *P* = 0.8520) between the six treatments (Table 7).

**Table 7.** Analysis of variance of mean percentage moisture content, weight loss, and germination of wheat infested with 25, 50, and 100 adults of *Sitophilus oryzae* subjected to hermetic storage treatment in 500-ml and 1,000-ml jars (*n* = 54)

Treatment	Parameter (%)*		
	Moisture content	Weight loss	Germination
25 insects/500 ml	7.50 ± 0.82	1.08 ± 0.13	93.33 ± 4.00
25 insects/1,000 ml	7.96 ± 0.21	0.81 ± 0.75	94.67 ± 4.00
50 insects/500 ml	8.37 ± 0.51	0.80 ± 0.23	92.44 ± 3.13
50 insects/1,000 ml	7.93 ± 0.85	0.81 ± 0.22	90.67 ± 3.46
100 insects/500 ml	7.77 ± 0.81	0.87 ± 0.39	94.67 ± 4.47
100 insects/1,000 ml	6.93 ± 1.02	0.62 ± 0.44	95.56 ± 5.07

*All data are means ± SEM. Entries in the same column were not significantly different (*P* ≥ 0.05). Means were separated using Bonferroni adjustment.

The germination capacity is the most sensitive to change during storage and was therefore used as an indicator of qualitative deterioration of stored wheat during storage. Before the trial, germination capacity was determined to be 92% for the wheat seeds. All treatment groups showed no statistically significant decline in germination after 28 d of storage relative to the initial measurements (*F*_5, 53_ = 1.75; *P* = 0.1421).

## Discussion

Effects of low O_2_ and high CO_2_ on *S. oryzae* (synonym *Calandra oryzae*) has been studied previously, with emphasis on exposure time to mortality (Bailey 1965). The studies found that substantial mortality is observed at 2% O_2_ (Calderon and Navarro 1980), as we observed for all treatments. Our study employed the use of acoustic technology to explore activity trends of *S. oryzae* in hermetic storage conditions. We found there was little insect activity after the O_2_ depleted to 5% and activity completely ceased after a level of 2%. Previous research has shown that O_2_ levels below 3% are most effective in controlling infestations (Navarro 1978, 2012; Moreno-Martinez et al. 2000). At farm level with hermetic bags like Purdue Improved Crop Storage (PICS) bags, O_2_ levels below 5% are achievable ((Baoua et al. 2014; Tubbs et al. 2016) and according to literature 5% also causes mortality but requires longer exposure time (Bailey 1955).

A focus of this study was the storage time needed to complete cessation of insect activity. We found that insects in 500-ml jars ceased activity in a short time compared with the larger 1,000-ml jars due to a lower amount of oxygen being available per insect for metabolism. We also found for the smallest population of 25 insects in 1,000 ml, that 13-d durations were needed for insect activity to cease, while 5 d were needed for the largest population in 500-ml jars. This was in agreement with findings that 6–9 d were taken to deplete O_2_ in *S. zeamais* infested maize grain (Moreno-Martinez et al. 2000). The insects were considered dead after burst rate fell below 0.02 bursts s^−1^. This was in agreement with Mankin et al. (2008), Jalinas et al. (2015), and Njoroge et al. (2016), who used 0.02 bursts s^−1^ as a threshold for low likelihood of infestation. Below this threshold burst level, the infestation was considered inconsequential due to less feeding and other damage to the grain. This implies that when hermetic storage treatment does not offer instant disinfestation, it may nevertheless render insects inactive and incapable of damaging the grain.

Analysis of insect activity using acoustic methods has been studied for several decades. Many of the studies focused on monitoring the effectiveness of acoustic detection for population density estimations, effects of temperature on insect activity, and the possibility of detecting hidden infestation (Kiobia et al. 2015; Njoroge et al. 2016, 2017). Our study focused on effects of O_2_ depletion on the activity of *S. oryzae* at different population densities. The results of this study have importance for farmers who store freshly harvested grain in PICS or similar bags. Freshly harvested grain may have a small infestation that multiplies to devastating numbers within 4–6 wk. The use of hermetic bags arrests this multiplication, and within a few weeks there is no more insect activity. The overall comparison showed that high-population density, i.e., 100 insects in 500 ml drove down the O_2_ level more rapidly than 25 insects in 1000 ml, with 3.1 d required for decrease to 5% and 5 d for decrease to 2% in the 100 insects/500 ml treatment, but 10.5 d for decrease to 5% and 13 d for 2% in the 25 insects/1,000 ml treatment.

Our regression results showed that the number of insects present as the starting population determines the decrease in sound activity as O_2_ declines. This observation is similar to what is observed with microbial death rate after heat treatment or treatment with antimicrobial agents.

The use of hermetic bags arrests insect population development and reduces insect feeding activity and insect damage. Our results show that farmers who use hermetic bags should not open them for at least 2–4 wk after they are filled to ensure there is sufficient time for oxygen depletion and elimination of insect activities.
